# Editorial: Factors for the progression from latent tuberculosis infection to tuberculosis disease, volume II

**DOI:** 10.3389/fcimb.2026.2007586

**Published:** 2026-10-13

**Authors:** Yushan Zhang, Chongguang Yang, Leonardo Martinez, Cheng Chen

**Affiliations:** 1 School of Public Health, Nanjing Medical University, Nanjing, Jiangsu, China; 2 Department of Chronic Communicable Disease Control and Prevention, Jiangsu Provincial Center for Disease Control and Prevention, Nanjing, Jiangsu, China; 3 Sun Yat-sen University, Guangzhou, China; 4 University of California, Berkeley, Berkeley, CA, United States

**Keywords:** active tuberculosis disease, host-directed therapy, immune reconstitution, latent tuberculosis infection, lipid metabolism, risk prediction model

According to the WHO report, there were approximately 10.8 million new tuberculosis cases and 1.25 million related deaths globally in 2024 (World Health Organization, 2025). The progression from latent tuberculosis infection (LTBI) to active tuberculosis disease (ATB) represents a critical bottleneck that constrains the achievement of global tuberculosis control targets. The lifetime risk of developing active disease ranges from 5% to 10% (Humphries, 2013; Menzies et al., 2021; Ekramnia et al., 2024). However, it is a complex pathological process jointly driven by multiple factors, including host immune status, pathogen biological strategies, environmental exposure, and sociodemographic characteristics (Ai et al., 2016; Horton et al., 2023; Litvinjenko et al., 2023). In this Research Topic, six research articles explored various perspectives, the molecular mechanisms underlying the conversion from LTBI to ATB, population-level risk characteristics, and novel intervention strategies. Collectively, they constructed a cross-scale analytical framework that spanned from macroscopic epidemiology to microscopic molecular mechanisms.

At the level of population risk identification and screening strategies, Xu et al. conducted a cross-sectional study of 1,184 PLWHA and found that middle-aged individuals aged 45–60 years and a CD4/CD8 ratio≥1 were significantly associated with QFT positivity. Notably, the CD4/CD8 ratio may not directly reflect the true risk of LTBI, but rather the improved sensitivity of QFT testing during immune reconstitution following ART. These findings have important implications for LTBI screening among PLWHA in low-TB-burden settings, emphasizing that interpretation of IGRA results should incorporate immune status to avoid missed diagnoses or misinterpretation based solely on numerical indicators.

Regarding risk prediction for progression in special populations, Lu et al. conducted a prospective cohort study focusing on close contacts in a school tuberculosis outbreak. They tracked 50 adolescent students and innovatively employed multiplex cytokine detection using QFT supernatant. By combining LASSO regression, they constructed a prediction model incorporating five indicators: QFT results, FGFbasic, IL-1ra, I-309/CCL1, and MPIF-1/CCL23. The model achieved an AUC of 0.939. This model offers a sensitive, specific risk stratification tool for high-risk adolescents, overcoming the low positive predictive value of conventional IGRA and enabling quantifiable precision TB control in schools.

At the level of regional epidemiology and environmental factors, Wang et al. conducted a large-scale population-based cross-sectional survey in the Tibet Autonomous Region. This study revealed the unique epidemiological characteristics of LTBI in high-altitude areas. The findings demonstrated that residential altitude was significantly negatively correlated with TST positivity rate. The prevalence at moderate altitudes (≤ 3500 m, 35.0%) was approximately 2.7 times that at very high altitudes (> 3500 m, 12.8%). This study incorporated environmental factors into the TB risk stratification framework. It suggests that the hypoxic environment at high altitudes may confer a protective effect through population sparsity and immunomodulation. However, smoking may synergistically exacerbate hypoxia-induced damage and eliminate this advantage.

At the level of molecular mechanism research, the review by Sundaram and Prabhu systematically summarized the roles of autophagy regulation, miRNA networks, and single-cell transcriptomics in the conversion from LTBI to ATB. *Mycobacterium tuberculosis* (*Mtb*) inhibited autophagy by activating the Rab1A–mTOR pathway through the PE_PGRS protein family. Multiple miRNAs targeted autophagy-related genes and promote intracellular bacterial survival. Single-cell sequencing had revealed differences in immune responses among CD4^+^ and CD8^+^ T cell subsets and NK cells at different stages of infection. The review concludes that the progression from LTBI to ATB is essentially a synergistic outcome of *Mtb*-driven autophagy and lysosome interference, miRNA dysregulation, and innate immune cell subset shifts. Risk stratification strategies may therefore provide key foundations for early TB warning and host-directed therapy.

Wang et al. focused on lipid metabolism as a central hub. They systematically elaborated the mechanisms of lipid metabolic reprogramming by *Mtb* during latent infection and reactivation. In the nutrient-limited microenvironment, *Mtb* initiated genome replication and enveloped remodeling through the MtrA regulator. It also achieved precise switching between lipid storage and utilization via the OmamC–Mce1 axis. At the host level, *Mtb* secreted 1-TbAd to effectively inactivate lysosomal function, thereby constructing a lipid-enriched metabolic microenvironment. The PPE2 protein further created conditions for distant dissemination by inducing systemic insulin resistance. This review proposes that multi-level intervention strategies targeting key nodes of lipid metabolism, in combination with host-directed therapy, may help overcome the clinical challenge of drug resistance and provide a novel pathway toward ending tuberculosis.

The last study introduced artificial intelligence technology into the front end of the tuberculosis diagnostic chain. Wang et al. developed a Siamese convolutional neural network model based on chest X-ray images to differentiate between aspergillosis, pneumonia, tuberculosis, and normal lungs. The model achieved a diagnostic accuracy of 98.72%. The study’s advances in radiological differentiation of active tuberculosis provide strong technical support for rapid triage of suspected patients in community screening and for shortening diagnostic delays. This helps improve closed-loop management from screening to confirmed diagnosis.

Synthesizing the six studies in this Research Topic, we can draw the following conclusions. The conversion from LTBI to ATB is a multi-stage and multi-dimensional process jointly shaped by host immune status, pathogen strategies, and environmental exposure. Precision intervention requires coordinated efforts at three levels. First, at the population level, multi-factor prediction models should be used for dynamic risk stratification of high-risk groups (Tingley et al., 2026). Second, at the mechanistic level, novel host-directed therapies should be developed by targeting key nodes such as the autophagy and lysosome pathway and lipid metabolism checkpoints. Third, at the technological level, artificial intelligence-based image interpretation should be integrated with multi-omics biomarkers to establish a full-chain precision diagnostic system. Whether the global goal of ending tuberculosis can be achieved depends fundamentally on our ability to translate such multi-dimensional risk knowledge into actionable and scalable primary care intervention strategies. The series of studies in this Research Topic provides important theoretical foundations and practical directions in this regard.
